# Supplementary material for: Programmable frequency-bin quantum states in a nano-engineered silicon device
Source: Nat Commun. 2023 Jan 12;14:176. doi: 10.1038/s41467-022-35773-6 (PMC9837142; doi:10.1038/s41467-022-35773-6)
Supplement: Supplementary file 1 — Supplementary Information [file 41467_2022_35773_MOESM1_ESM.pdf]

Supplementary Information for: Programmable frequency-bin quantum states in a nano-engineered silicon device

Supplementary Note 1 - Experimental setup

The experimental setup used for the measurements in this work is shown in Supplementary Figure 1. A tunable laser was coupled through a lensed fiber (SMF-28, 3 µm mode field diameter) to the sample, either at the input arm of the MZI (Φ configuration), or at the bus waveguide (Ψ configuration and linear spectroscopy). add The MZI was designed so that, with no bias applied, the pump is routed to the output on the top as represented in Fig. 6 of the main text. A band-pass filter (BP) centred at the frequency of the laser was used to increase the side mode suppression, while the polarization was defined through a fiber polarization controller (PC). An electro-optic phase modulator (EOM) was included before the chip input in order to tailor the pump spectrum for operation in the Ψ configuration. The overall device footprint is around 0.026 mm^2 if operated in Φ configuration, and 0.007 mm^2 if operated

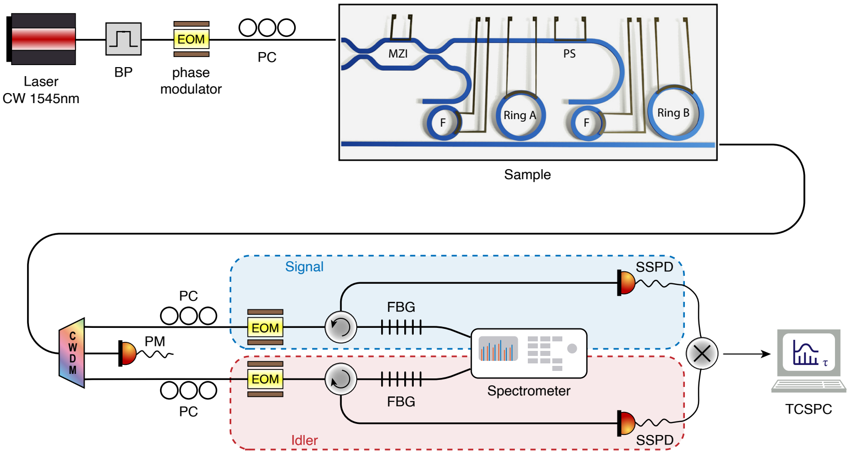

Supplementary Figure 1 Experimental setup.

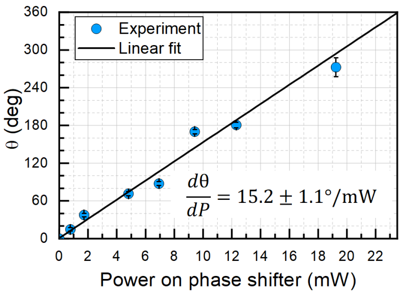

Supplementary Figure 2 Estimated phase of the generated state $\Phi(\theta)$, as defined by Eq. (1), as a function of the electrical power dissipated on the PS. Each experimental point is estimated from best fit of a quantum interference curve. Error bars represent the error associated to each fit.

in Ψ configuration (i.e. excluding the MZI and the filter rings). The generated photons were collected using a second lensed fiber, and a telecom-grade Coarse Wavelength Division Multiplexer (CWDM) was used us to spectrally separate the residual pump laser (1540 nm-1560 nm) - which was monitored by an InGaAs power-meter (PM) - from the idler (1560 nm-1580 nm) and signal (1520 nm-1540 nm) photon bands with low inter-channel crosstalk (< -80 dB). In order to select the frequency bins of interest and to separate them for the residual SFWM signal, two narrowband fiber Bragg gratings (FBGs), in combination with optical circulators, were employed. These filters can be tuned, and this enables the selective routing of the photons generated within the different resonances to superconductive single-photon detectors (SSPD). The FBGs stop-band of approximately 8 GHz allowed us to precisely select the bin of interest for modulation frequencies $f_{\mathrm{m}} > 4$ GHz, and at the same time prevented any unwanted photon outside this band from reaching the detectors. An additional pair of amplitude EOMs was exploited to manipulate the generated non-classical state, while a spectrometer was used to monitor the SFWM spectrum coming from the sample. Finally, the electrical signal coming from the SSPDs was recorded and analyzed through a time-tagging electronic system, where time-correlated single-photon counting (TCSPC) was performed.

Phase shifter

To control the phase of the generated state in the Φ configuration, we operated a thermo-optic phase shifter (PS) affecting the relative phase of the pump field driving Ring B with respect to the one driving Ring A. The suitability of this technique was assessed by probing the phase of the quantum interference curve as a function of the electrical power applied to the phase shifter, as shown if Supplementary Figure 2. Deviations from the linear trend are attributed to thermal and electrical crosstalk, which can be mitigated by an active feedback loop acting on the thermo-optic actuators.

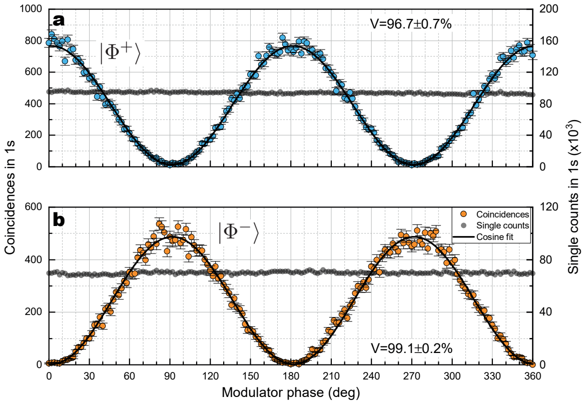

Supplementary Figure 3 Blue and red dots: Bell curve measurements for the states $|\Phi^+\rangle$ (panel **a**) and $|\Phi^-\rangle$ (panel **b**), left axis. Error bars are calculated assuming Possonian statistics. Grey dots: single counts (two-detectors average), right axis.

Supplementary Note 2 - Generated states and quantum interference

In order to assess the entangled nature of the quantum states generated, we set $f_\mathrm{m} = \Delta/2$ and varied φ_s to perform a Bell-like experiment. Two examples of the resulting quantum interference curves are shown in Supplementary Figure 3a-b, corresponding respectively to $\theta = 0$ (i.e., the state $|\Phi^+\rangle$) and $\theta = \pi$ (i.e., the state $|\Phi^-\rangle$) as given in Eq. (1). Each point of the interference curves for states generated in the Φ configuration represents the outcome of a positive operator-valued measurement (POVM) performed on the generated state ρ:

$$R(\alpha) = R_0 \operatorname{tr}(\rho \, |\Phi(\alpha)\rangle \langle\Phi(\alpha)|), \tag{1}$$

where R is the coincidence rate, and $|\Phi(\alpha)\rangle = (|00\rangle + e^{i\alpha}|11\rangle)/\sqrt{2}$. An analogous definition holds for states generated in configuration Ψ, where the projection state is replaced by $|\Psi(\alpha)\rangle = (|01\rangle + e^{i\alpha}|10\rangle)/\sqrt{2}$.

The curves of the coincidence measurements clearly show the expected interference, while single counts do not show any visible interference pattern; entanglement is verified if the interference visibility exceeds $1/\sqrt{2}$ [1]. In our case, the visibilities for the $|\Phi^+\rangle$ and $|\Phi^-\rangle$ states are $96.7 \pm 0.7\%$ and $99.7 \pm 0.2\%$, respectively, confirming entanglement with a degree of confidence exceeding 140 standard deviations in the latter case. When compared to the literature, this is a significant value for a Bell test, and a remarkable achievement when compared with other results obtained using frequency-bin encoding [2]. A time-resolved version of the above curves for each of the states considered in this work is also presented in Supplementary Figure 4.

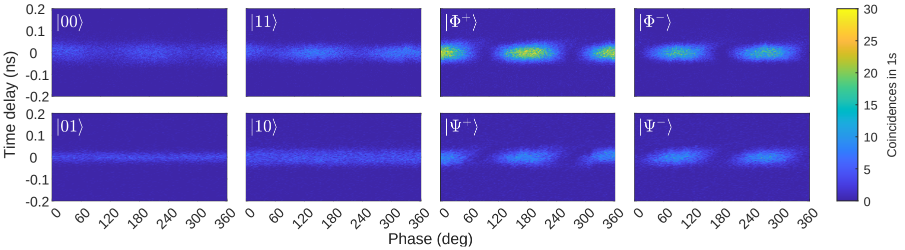

Supplementary Figure 4 Quantum interference curves observed as a function of the phase applied on the EOM at the idler channel. Each vertical slice represents a coincidence histogram as shown in Fig. 2a inset of the main text.

Supplementary Note 3 - Quantum interference between two frequency bins

We consider the case in which photon pairs are generated via single-pump SFWM in a coherent superposition of two frequency bins. The biphoton wavefunction describing the photon pairs can be written as:

$$\phi(\omega_1,\omega_2) = \phi_A(\omega_1,\omega_2) + e^{i\theta}\phi_B(\omega_1,\omega_2), \tag{2}$$

where $\phi_{A(B)}(\omega_1,\omega_2)$ is (up to a normalization factor) the biphoton wavefunction associated with the bin A(B), and θ is the phase difference between the two bins, which we assume to be independent of frequency.

As usual, the biphoton wavefunction is symmetric with respect to the exchange of ω_1 and ω_2. For simplicity, in the following we focus on the portion of the (ω_1,ω_2) corresponding to the case $\omega_1 < \omega_\mathrm{p}$ and $\omega_2 > \omega_\mathrm{p}$, with ω_p the central frequency of a generic pump pulse, with $\omega_{1(2)}$ indicating the signal (idler) photons.

We assume that the generated photons are separated in idler and signal, sent through two electro-optic modulators (EOMs), and detected in a coincidence experiment. In particular, we look at the coincidence events in the portion of the spectrum in which the two bins can interfere, with

$$\tilde{\phi}_A(\omega_1,\omega_2) = g_A(\omega_1 - \omega_{A,\mathrm{s}} + \omega_\mathrm{m}, \omega_2 - \omega_{A,\mathrm{i}} - \omega_\mathrm{m})e^{i(\omega_2-\omega_{A,\mathrm{i}}-\omega_\mathrm{m})\delta T} \tag{3}$$

and

$$\tilde{\phi}_B(\omega_1,\omega_2) = e^{i\theta}g_B(\omega_1 - \omega_{B,\mathrm{s}} - \omega_\mathrm{m}, \omega_2 - \omega_{B,\mathrm{i}} + \omega_\mathrm{m})e^{i(\omega_2-\omega_{B,\mathrm{i}}+\omega_\mathrm{m})\delta T}, \tag{4}$$

the biphoton wavefunctions of the converted photon pairs. Here $(\omega_{A(B),\mathrm{s}}, \omega_{A(B),\mathrm{i}})$ is the center of the $A(B)$ bin before the conversion, $\omega_\mathrm{m}/2\pi = f_\mathrm{m}$ is the EOM modulation frequency, and δT a generic delay due to a path difference between signal and idler before the EOM. Finally, $g_{A(B)}(\omega_1,\omega_2)$ is a function centred in the origin and describing the frequency correlation of the original bin $A(B)$. Then the biphoton wavefunction

describing the detected pairs can be written as:

$$\tilde{\phi}(\omega_1, \omega_2) = \tilde{\phi}_A(\omega_1, \omega_2) + \tilde{\phi}_B(\omega_1, \omega_2). \tag{5}$$

Without loss of generality we take $(\omega_{B,\mathrm{s}} - \omega_{A,\mathrm{s}})/2\pi = (\omega_{A,\mathrm{i}} - \omega_{B,\mathrm{i}})/2\pi = \Delta$, and we write:

$$\begin{aligned}\tilde{\phi}(\omega_1, \omega_2) &= g_A(\omega_1 - \omega_{A,\mathrm{s}} + \omega_\mathrm{m}, \omega_2 - \omega_{A,\mathrm{i}} - \omega_\mathrm{m}) e^{i(\omega_2 - \omega_{A,\mathrm{i}} - \omega_\mathrm{m})\delta T} \\ &+ g_B(\omega_1 - \omega_{A,\mathrm{s}} + 2\pi\Delta - \omega_\mathrm{m}, \omega_2 - \omega_{A,\mathrm{i}} - 2\pi\Delta + \omega_\mathrm{m}) e^{i((\omega_2 - \omega_{A,\mathrm{i}} - 2\pi\Delta + \omega_\mathrm{m})\delta T + \theta)},\end{aligned} \tag{6}$$

with the generation rate given by:

$$\begin{aligned}R_\mathrm{gen}(\omega_\mathrm{m}) &= \int d\omega_1 d\omega_2 \mid \tilde{\phi}(\omega_1, \omega_2) \mid^2 \\ &= \int d\Omega_1 d\Omega_2 \mid g_A(\Omega_1, \Omega_2) \\ &+ g_B(\Omega_1 + 2(\pi\Delta - \omega_\mathrm{m}), \Omega_2 - 2(\pi\Delta - \omega_\mathrm{m})) e^{i(\theta - 2(\pi\Delta - \omega_\mathrm{m})\delta T)} \mid^2 \\ &= \int d\Omega_1 d\Omega_2 \mid g_A(\Omega_1, \Omega_2) \mid^2 \\ &+ \int d\Omega_1 d\Omega_2 \mid g_B(\Omega_1 + 2(\pi\Delta - \omega_\mathrm{m}), \Omega_2 - 2(\pi\Delta - \omega_\mathrm{m})) \mid^2 \\ &+ 2 \int d\Omega_1 d\Omega_2 \mathrm{Re}\left[g_A^*(\Omega_1, \Omega_2) \right. \\ &\times \left. g_B(\Omega_1 + 2(\pi\Delta - \omega_\mathrm{m}), \Omega_2 - 2(\pi\Delta - \omega_\mathrm{m})) e^{i(\theta - 2(\pi\Delta - \omega_\mathrm{m})\delta T)} \right].\end{aligned}$$

A very simple case

If we consider identical bins, with $g_A(\Omega_1, \Omega_2) = g_B(\Omega_1, \Omega_2)$ and $\omega_\mathrm{m}/2\pi = \Delta/2$, that is a modulation frequency equal to half of the bin frequency separation, one has immediately

$$R_\mathrm{gen}(\Delta) = 2\left[1 + \cos(\theta)\right] \int d\Omega_1 d\Omega_2 \mid g_A(\Omega_1, \Omega_2) \mid^2, \tag{7}$$

which means that we have perfect interference between the two bins. The result of the interference depends on the value of the phase θ.

A simple case

We consider, identical bins but a generic modulation frequency. In this case we have

$$R_\mathrm{gen}(\Delta) = 2 \int d\Omega_1 d\Omega_2 \mid g_A(\Omega_1, \Omega_2) \mid^2 \tag{8}$$

$$+\ 2\int d\Omega_1 d\Omega_2 \mathrm{Re}\left[g_A^*(\Omega_1,\Omega_2)\right.$$
$$\left.\times\ g_A(\Omega_1+2(\pi\Delta-\omega_{\mathrm{m}}),\Omega_2-2(\pi\Delta-\omega_{\mathrm{m}}))e^{i(\theta-2(\pi\Delta-\omega_{\mathrm{m}})\delta T)}\right].$$

If we consider the case of a very narrow pump (i.e. quasi-CW regime) centred at $\omega_{\mathrm{p}}=(\omega_{A,\mathrm{i}}+\omega_{A,\mathrm{s}})/2$ and a ring resonator with Lorentian resonances having full width at half maximum $\gamma/2\pi=\Gamma$, one can write (see Ref.[3])

$$g(\omega_1,\omega_2)\approx G\,\frac{1}{\omega_1-i\gamma/2}\,\frac{1}{\omega_2-i\gamma/2}\,\mathrm{sinc}\left(\frac{\omega_1+\omega_2}{\Delta\omega}\right), \tag{9}$$

where G is a constant, and $\Delta\omega\ll\gamma$ is the spectral pump width.

By inserting (9) in the second term of Eq. (8), one gets (after a bit of algebra and approximating the sinc^2 function with a delta function):

$$\begin{aligned}
&2\int d\Omega_1 d\Omega_2 \mathrm{Re}\left[g_A^*(\Omega_1,\Omega_2)g_A(\Omega_1+2(\pi\Delta-\omega_{\mathrm{m}}),\Omega_2-2(\pi\Delta-\omega_{\mathrm{m}}))e^{i(\theta-2(\pi\Delta-\omega_{\mathrm{m}})\delta T)}\right]\\
&=2\left|G\right|^2\mathrm{Re}\left[e^{i(\theta-2(\pi\Delta-\omega_{\mathrm{m}})\delta T)}\right]\int d\Omega_1\frac{1}{\Omega_1^2+\frac{\gamma^2}{4}}\,\frac{1}{(\Omega_1-2(\pi\Delta-\omega_{\mathrm{m}}))^2+\frac{\gamma^2}{4}}\\
&=\left|G\right|^2\frac{\gamma}{4\pi}\,\frac{1}{(\omega_{\mathrm{m}}-\pi\Delta)^2+\gamma^2}\cos\left(\theta-2(\omega_{\mathrm{m}}-\pi\Delta)\delta T\right),
\end{aligned} \tag{10}$$

$$R=A\left[1+\frac{\Gamma^2}{(f_{\mathrm{m}}-\Delta/2)^2+\Gamma^2}\cos\left(4\pi(f_{\mathrm{m}}-\Delta)\delta T-\theta\right)\right]. \tag{11}$$

Supplementary References

[1] Clauser, J.F., Horne, M.A., Shimony, A., Holt, R.A.: Proposed experiment to test local hidden-variable theories. Phys. Rev. Lett. **23**, 880–884 (1969). https://doi.org/10.1103/PhysRevLett.23.880

[2] Imany, P., Jaramillo-Villegas, J.A., Odele, O.D., Han, K., Leaird, D.E., Lukens, J.M., Lougovski, P., Qi, M., Weiner, A.M.: 50-GHz-spaced comb of high-dimensional frequency-bin entangled photons from an on-chip silicon nitride microresonator. Optics Express **26**(2), 1825 (2018). https://doi.org/10.1364/oe.26.001825

[3] Onodera, T., Liscidini, M., Sipe, J., Helt, L.: Parametric fluorescence in a sequence of resonators: An analogy with dicke superradiance. Physical Review A **93**(4), 043837 (2016). https://doi.org/10.1103/PhysRevA.93.043837
